# Inhaled Corticosteroids Use Is Not Associated With an Increased Risk of Pregnancy-Induced Hypertension and Gestational Diabetes Mellitus: Two Nested Case-Control Studies: Erratum

**DOI:** 10.1097/01.md.0000494752.13423.51

**Published:** 2016-08-26

In the article “Inhaled Corticosteroids Use Is Not Associated With an Increased Risk of Pregnancy-Induced Hypertension and Gestational Diabetes Mellitus: Two Nested Case-Control Studies”,^1^ which appeared in Volume 95, Issue 22 of *Medicine*, one of Dr. Jimin Kim's affiliations was originally omitted.

The article has since been corrected online.
